# Predictive Factors for Postoperative Outcome in Children with Jejunoileal Atresia

**DOI:** 10.1055/s-0039-1697628

**Published:** 2019-10-01

**Authors:** Charlotta Jarkman, Martin Salö

**Affiliations:** 1 Department of Pediatric Surgery, Skåne University Hospital, Lund, Sweden; 2 Department of Clinical Sciences, Pediatrics, Lund University, Lund, Sweden

**Keywords:** jejunoileal atresia, outcome, complication

## Abstract

**Background** Jejunoileal atresia is a relatively rare congenital gastrointestinal requiring surgery and long postoperative care. The postoperative outcome is affected by many factors and this study focuses on finding predictors for time to full enteral feeding, length of hospital stay (LOH), and postoperative complications.

**Methods** This was a retrospective study of all children operated for isolated jejunoileal atresia between 2001 and 2017 at a tertiary center of pediatric surgery. Independent variables regarding demographical-, operative-, and postoperative data were abstracted. Primary outcome was time to full enteral feeding, LOH, and postoperative complications in terms of reoperation or central line complication. Any significant variables from the univariate analysis were further analyzed with logistic regression and presented as odds ratio with 95% confidence interval.

**Results** After exclusion because of concomitant gastroschisis ( *n* = 1), and death before discharge ( *n* = 2), 47 patients were further analyzed (49% boys, 53% premature). No significant differences could be seen in the univariate analysis between children with short and long time (median > 17 days) to full enteral nutrition. Patients with longer LOH (median >32 days) had significantly lower birth weight compared with those with shorter LOH; median 2,550 g versus 2,980 g ( *p* = 0.04). Patients with a central line complication had significantly longer median time to full enteral feeding (median 27 vs. 12 days, *p* = 0.03), and significantly longer median LOH (median 43 vs. 21 days, *p* = 0.03), but these parameters were not significant in a multivariate analysis. No significant results were found regarding reoperation.

**Conclusion** Low birth weight seems associated with an increased LOH in children operated on for jejunoileal atresia, and central line complications seem related to the duration with central line in this group. The small cohort may constitute a power problem in this study and further research regarding the included variables may reveal more potential predictors for the postoperative outcome.

Jejunoileal atresia (JIA) is a congenital gastrointestinal defect with a birth prevalence of ∼1/5,000 to 1/14,000 live births. 1 2 3 4 5 The main postoperative goal is to initiate and to reach, if possible, full enteral nutrition while avoiding postoperative complications.

The time to full enteral nutritional intake varies greatly within the group of JIA, and it is mainly for this reason they remain at the hospital. There are a few known factors affecting the time to full enteral intake and hence, length of hospital stay (LOH). Prior studies have shown that time to full enteral feeding is affected by type (jejunal or ileal) of JIA, remaining bowel length, 6 7 8 presence of short bowel syndrome, 9 and other cooccurring birth defects. 10 Postoperative complications are common in children with JIA at least one in five experiences a complication requiring an operative intervention. 9 11 Complications may of course have adverse effects on the enteral or parenteral nutrition and increase in LOH.

Today, 84 to 90% of infants with atresia survive and most of them have a normal development. 11 12 13 Considering the favorable outcome these children often have, it is of great importance to investigate what factors can hinder this by adding to the risk of complications and to an increased duration to full enteral feeding and LOH, and in the long run result in increased morbidity and even death. Therefore, the aim of this study was to investigate predictive factors for time to full enteral feeding, LOH, and postoperative complications in children operated on for JIA. The information from this study could be used for planning and improvement in the care of these children, as well as to provide important data that could be used in parental information prenatally, preoperatively, and postoperatively.

## Material and Method

The study was approved by the regional ethical board (DNR no 2010/49).

### Settings and Children

All children were treated at a tertiary center for pediatric surgery with a catchment area of ∼1.8 million inhabitants for specialized pediatric surgery in children up to 15 years. All patients from 2001 through 2017 with international classification of diseases (ICD-10) diagnosis code Q40.1–2 or Q40.8–9 were eligible for inclusion. The inclusion criterion was operation for isolated JIA, and exclusion criterion was any major concomitant gastrointestinal anomaly or death before discharge from hospital since this would affect the primary outcomes.

### Study Design

This is a retrospective study of all patients admitted to a tertiary center of pediatric surgery between 2001 and 2017 for JIA. Primary outcome was time to full enteral feeding, LOH, and postoperative complications in terms of reoperation or central line complication.

Independent variables for the primary outcomes were gender, prematurity, small for gestational age, birth weight, cardiac anomaly, type of atresia and/or residual bowel length, and primary anastomosis or stoma. For LOH, complication grade was also added, and for central line complications days until full enteral nutrition and LOH were added as independent variables. Medical and surgical journals were reviewed and one researcher abstracted all data. For all calculations, the cohort was dichotomized. Regarding time to full enteral feeding and LOH, the cohort was divided by the median time (for each parameter) for the whole cohort, due to the wide range of these two primary outcomes (and hence, a linear regression was not suitable). Regarding the complications (reoperation and central line complication), the cohort was divided by presence or absence of the specific complication.

### Definitions and Classification

Prematurity was defined as gestational week < 37. Prenatal signs on ultrasound included any sign that led to believe there could be an obstruction somewhere along the intestine such as dilated loops or polyhydramnios. Time to full enteral nutrition was calculated from the day of surgery until full enteral feeding without any parenteral nutrition. Enteral feeding included feeding per os, nasogastric feeding tube, and jejunal feeding tube. In LOH, children who went home on parenteral nutrition were still counted as discharged. In reoperations, a planned take down of a stoma was not included even if it occurred before discharge. Complications were divided into grade I to IV according to the Clavien-Dindo classification. Grade I included complications not requiring any treatment, grade II complications requiring pharmacological treatment, grade III complications requiring procedures with anesthesia, and grade IV life-threatening complications.

### Statistical Analyses

Data analyzed with IBM SPSS Statistics for Mac, version 24. Dichotomous variables were presented as the absolute number and percentage of patients, *n* (%), and analyzed with Fisher's exact test. Continuous variables were presented as median (min-max) and analyzed with Mann–Whitney U test. Significant variables in the univariate analysis, if any, were further analyzed with multivariable logistic regression and presented as odds ratio (OR) with 95% confidence interval (CI). Continuous parameters were logarithmized in the regression model due to no normal distribution. A *p* -value < 0.05 was considered significant.

## Results

**Fig. 1:**
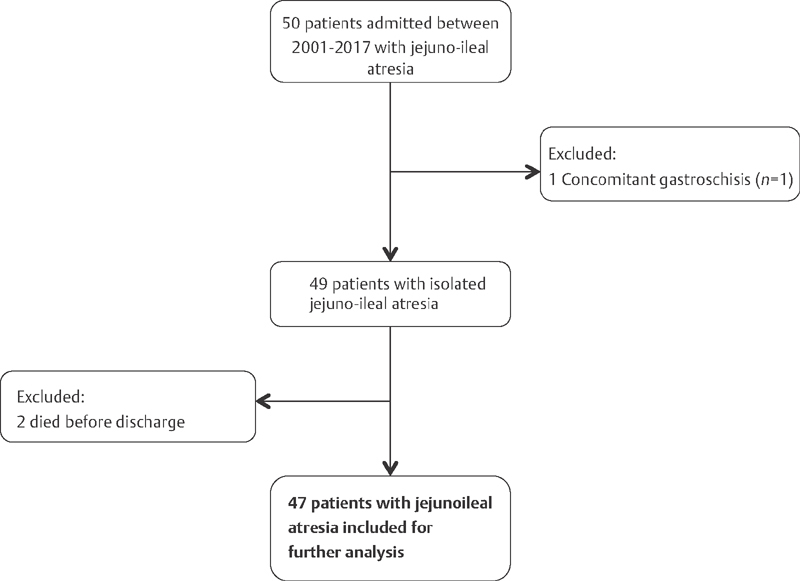
Flowchart of inclusion and exclusion of children with jejunoileal atresia between the years 2001 to 2017.

A total of 50 patients were eligible for inclusion. Of these, one patient was excluded because of concomitant gastroschisis, and two patients were excluded because they died of sepsis before discharge, leaving 47 patients for further analysis ( Fig. 1 ). Of the included children, 23 (49%) were males and 25 (53%) were born prematurely, and the median birth weight was 2761 (1,590–4,425) g. Prenatally detected signs of obstruction on ultrasound were found in 23 infants (49%). A congenital heart defect was found in five (11%) patients ( Table 1 ).

**Table 1:** Preoperative and operative data in 47 children operated on for jejunoileal atresia

Preoperative data	
Gender (M/F)	23 (49%)/24 (51%)
Twin	3 (6%)
Prenatal signs on ultrasound	23 (49%)
Premature (GW < 37)	25 (53%)
Gestational week	35 + 0 (33 + 2–36 + 5)
SGA	2 (4%)
Birth weight (grams)	2761 (1,590–4,425) ^a^
Other anomalies	
GI anomaly	8 (17%)
Cardiac anomaly	5 (11%)
Cleft palate	1 (2%)
Urinary tract anomaly	1 (2%)
Cystic fibrosis	2 (4%)
Operative data	
Age at operation (days)	1 (0–8)
Type of atresia *	
Type 1	11 (24%)
Type 2	13 (28%)
Type 3a	8 (17%)
Type 3b	7 (15%)
Type 4	7 (15%)
Location of atresia	
Jejunum	26 (55%)
Ileum	21 (45%)
Primary anastomosis	33 (70%)
Resection of intestine	39 (83%)
Stoma	
Double barrel stoma	17 (36%)
Jejunostomy	1 (2%)
Ileostomy	1 (2%)
Residual length of small bowel (cm)	87 (15–230) ^b^
Resected ileocecal valve	2 (4%)

Values presented as absolute numbers and percentage of patients; *n* (%) and median (min-max); F, female; GI, gastrointestinal; GW, gestational week; M, male; SGA, small for gestational age.

* Total number of different types of atresia in 43 patients; lacking data for ^a^ 1 patient, ^b^ 20 patients.

The type and location of the atresia were equally distributed among type I to IV and jejunum/ileum, respectively. A majority of the patients had the bulbous end of the intestine resected, 39 (83%). A primary anastomosis was performed in 33 (70%) of the patients, while 19 (40%) patients received a primary stoma; a few patients had multiple atresias and hence, had both an anastomosis and a stoma. The median residual length of small bowel was 87 (15–230) cm; however, 20 (43%) patients lacked data ( Table 1 ).

Time to full enteral nutrition postoperatively was a median 17.5 (4–242) days. Four (10%) infants were discharged with parenteral nutrition. Children with ileal atresia reached enteral autonomy faster than those with jejunal atresia (median 10.5 vs. 20 days, *p* = 0.04).

The overall complication rate was 66%, with the majority having a grade II- or III complication according to the Clavien-Dindo classification. Central line complications (infection, thrombosis, dysfunction) and sepsis were the most common complications. The median LOH was 32 (10–436) days ( Table 2 ) and did not differ between children with ileal versus jejunal atresia (22 vs. 34 days, *p* = 0.10).

**Table 2:** Postoperative data and outcome in 47 children with jejunoileal atresia

Postoperative data	
PICU (days)	3 (0–99)
Central line/PICC	25 (53%)/24 (51%)
Nasogastric feeding tube	45 (96%)
Jejunal feeding tube	11 (23%)
Start of enteral nutrition postoperatively (days)	2 (0–22) ^b^
Nasogastric tube feeding (days)	16.5 (3–105) ^a^
Days until full enteral nutrition	17.5 (4–242) ^c^
Discharge with parenteral nutrition	4 (10%) ^d^
Any complication	31 (66%)
Complications ( *n* and percentage of complications)	
Grade I	3 (7%)
Cholestasis	
Grade II	17 (40%)
Sepsis (6), central line thrombosis (4), central line infection (6), wound infection, central line dysfunction (3), pneumonia	
Grade III	20 (46%)
Central line dysfunction (5), central line infection (3), central line thrombosis, intestinal obstruction (new stoma), intestinal hematoma, intestinal obstruction (2), revision of stoma, intestinal stricture (2), narrow stoma (2), subacute serial transverse enteroplasty, wound rupture, anastomotic insufficiency (2), prolapse of stoma	
Grade IV	3 (7%)
Sepsis (2), endocarditis	
LOH	32 (10–436) ^g^
Discharged to	
Home	35 (75%)
Other hospital	12 (25%)

Values presented as absolute numbers and percentage; *n* (%) and median (min-max); BF, breast feeding; IV, intravenous; LOH, length of stay at hospital; NG, nasogastric; PICC, peripherally inserted central catheter; PICU, pediatric intensive care unit; PN, total parenteral nutrition; PO, per os; Sub/supp, milk substitute/supplement; lacking data for ^a^ : 21 patients; ^b^ : 1 patient; ^c^ : 11 patients; ^d^ : 7 patients; ^e^ : 12 patients; ^f^ : 12 patients; ^g^ : 12 patients.

Comparing children with short versus long time to full enteral feeding, no differences could be found regarding gender, prematurity, small for gestational age, birth weight, cardiac anomaly, type of atresia, primary anastomosis, or residual bowel length ( Table 3 ). In the group with longer hospital stay, there was a significantly lower median birth weight (median 2,550 vs. 2,980 g) ( *p* = 0.04), while no differences could be seen regarding gender, prematurity, small for gestational age, birth weight, cardiac anomaly, type of atresia, primary anastomosis, complication grade ≥2, or residual bowel length ( Table 4 ).

**Table 3:** Parameters associated with shorter and longer time to full enteral nutrition

	≤ 17 days ( *n* = 18)	> 17 days ( *n* = 18)	*p* -Value
**Gender** (male)	10 (56%)	9 (50%)	1 *
Premature (<GW 37)	6 (33%)	12 (67%)	0.09 *
SGA	0 (0%)	2 (11%)	0.49 *
Birth weight (g)	3,182 (2,040–4,425)	2,655 (1,590–4,175)	0.09 **
Cardiac anomaly	3 (17%)	2 (11%)	1 *
Type of atresia (jejunum)	7 (39%)	11 (61%)	0.31 *
Primary anastomosis	12 (67%)	13 (72%)	1 *
Residual length of small bowel (cm)	155 (70–250)	85 (35–250)	0.13 *

Values presented as absolute numbers and percentage; *n* (%) and median (min-max); GW: gestational week; SGA: small for gestational age.

* Fisher's exact test.

** Mann–Whitney U test.

**Table 4:** Parameters associated with longer or shorter length of stay at hospital

	≤ 32 days ( *n* = 18)	> 32 days ( *n* = 17)	*p* -Value
Gender (male)	11 (61%)	6 (33%)	0.18 *
Premature (<GW 37)	7 (39%)	12 (71%)	0.09 *
SGA	0 (0%)	2 (12%)	0.23 *
Birth weight (g)	2980 (2,040–4175)	2550 (1,590–4,070)	0.04 **
Cardiac anomaly	2 (11%)	3 (18%)	0.66 *
Type of atresia (jejunum)	8 (44%)	12 (71%)	0.18 *
Primary anastomosis	12 (67%)	12 (71%)	1 *
Complication gr ≥ 2	7 (39%)	11 (65%)	0.18 *
Residual length of small bowel (cm)	160 (70–250) ^a^	80 (15–250) ^b^	0.06 **

Values presented as absolute numbers and percentage; *n* (%) and median (min-max); LOH, length of hospital stay; SGA, small for gestational age.

* Fisher's exact test.

** Mann-Whitney U test. Lacking data for ^a^ : 9 patients, ^b^ : 3 patients.

Regarding postoperative complications, no significant differences could be found between children with and without a reoperation ( Table 5 ). Ten patients needed a reoperation because of intestinal obstruction (3), revision of stoma, intestinal stricture (2), wound rupture, anastomotic insufficiency (2), intestinal hematoma, and subacute serial transverse enteroplasty (one patient needed more than one operation). There was no difference in rate of reoperations between children with ileal and jejunal atresia (19 vs. 23%, *p* = 1).

**Table 5:** Parameters associated with reoperation < 30 days postoperatively in 47 children with jejunoileal atresia

	Reoperation ( *n* = 10)	No reoperation ( *n* = 37)	*p* -Value
Gender (M)	5 (50%)	18 (49%)	1 *
Premature (GW < 37)	5 (50%)	20 (54%)	1 *
Birth weight (g) ^a^	2952 (2,280–3,895)	2,696 (1,590–4,425)	0.258 **
Cardiac anomaly	1 (10%)	4 (11%)	1 *
Type of atresia (jejunum)	7 (70%)	19 (51%)	0.475 *
Primary anastomosis	5 (50%)	28 (76%)	0.137 *
Residual length of small bowel (cm) ^b^	110 (25–166)	100 (15–260)	0.596 **

Values presented as absolute numbers and percentage; *n* (%) and median (min-max); GW: gestational week; lacking data for ^a^ : 1 patient; ^b^ : 20 patients.

* Fisher's exact test.

** Mann–Whitney U test.

When evaluating the occurrence of a central line complication, time to full enteral feeding (median 27 vs. 12 days) ( *p* = 0.027), and LOH (median 43 vs. 21 days) ( *p* = 0.032), was significantly longer in children with a central line complication ( Table 6 ). In the multivariate analysis, with time to full enteral feeding and LOH as independent variables, no significant results were found: OR 1.85 (95% CI: 0.52–6.5) ( *p* = 0.342) and OR 3.06 (95% CI: 0.78–11.96) ( *p* = 0.108), respectively. There was no difference in rate of central line complications between children with ileal and jejunal atresia (29 vs. 42%, *p* = 0.38).

**Table 6:** Parameters associated with central line complications in 47 children with jejunoileal atresia

	Complication ( *n* = 17)	No complication ( *n* = 30)	*p* -Value
Gender (M)	6 (35%)	17 (57%)	0.227 *
Premature (GW < 37)	12 (71%)	13 (43%)	0.127 *
Birth weight (g) ^a^	2,565 (2,100–4,070)	2,962 (1,590–4,425)	0.204 **
Cardiac anomaly	2 (12%)	3 (10%)	1 *
Stoma	8 (47%)	11 (37%)	0.769 *
Residual length of small bowel (cm) ^b^	75 (15–260)	110 (25–260)	0.121 **
Days until full enteral nutrition ^c^	27 (13–242)	12 (4–59)	0.027 **
LOH (days) ^d^	43 (17–254)	21 (10–106)	0.032 **

Values presented as absolute numbers and percentage; n (%) and median (min-max); GW, gestational week; LOH, length of hospital stay; lacking data for ^a^ : 1 patient; ^b^ : 20 patients, ^c^ : 11 patients, ^d^ : 12 patients.

* Fisher's exact test.

** Mann–Whitney U test.

## Discussion

In this retrospective cohort study of children with JIA, lower birth weight was associated with longer hospital stay, and children with a postoperative central line complication had significantly longer hospital stay and significantly longer time to full enteral feeding compared with children without a complication.

Enteral nutrition is a major part of the postoperative treatment and factors related to this, positive or negative, are important to investigate. However, no differences were found when comparing infants with longer and shorter time to full enteral nutrition. The small cohort and hence, lack of power, could explain this, and our results may indicate that prematurity and birth weight may be contributing factors, and hence could be potentially interesting variables in future studies. Our study showed a median time to full enteral nutrition of 17.5 days, which is similar to results found in other studies. 6 7 9 10 For example, one study showed a median time to full enteral feeding ranging from 8 to 20 days with the antenatally diagnosed cases taking longer time. 6 Another study regarding postoperative outcome found an average of 2 to 3 weeks to full enteral nutrition; however, infants with short-bowel syndrome required significantly longer time of parenteral nutrition than those with normal bowel length (49 vs. 16 days). 7 Other studies have shown a mean time of 3 weeks with significant correlation to residual bowel length, 9 and co-occurring birth anomalies. 10

When evaluating LOH, a significantly lower median birth weight was found in the group with longer hospital stay. This is not a surprising result, although it has never been described in the literature before. Studies have, however, shown that low birth weight is related to higher mortality in patients with JIA. 7 14 Overall, the results suggest that low birth weight might be related to a more severe postoperative period.

The overall complication rate in our cohort was 66%, considerably higher than 18 to 46% found in other studies. 9 10 11 15 The difference in complication rate could be explained by the amount of different complications taken into account, and the present study included central line complications compared with previous studies. Hence, the most common complications in this study were central line complications, including infection, thrombosis, and dysfunction. Infants with any central line complication had significantly longer time to full enteral nutrition as well as significantly longer stay at the hospital. With a central line complication, time with parenteral nutrition is often prolonged, and there is often a need for a surgical intervention under general anesthesia, which temporarily disrupts and prolongs time to full enteral nutrition and hence, it lengthens hospital stay. However, the causality could also be the other way around; the longer the infants need to stay on parenteral nutrition or in the hospital, the higher the risk of acquiring a central line complication.

The weakness of this study is the retrospective data collection, the small study population, and in some instances the lack of data further decreased the number of patients being analyzed. It could explain the low number of significant results. To have results with higher statistical power, further studies with larger study populations are needed. Even though this study was small demographically, it is congruent with other studies regarding JIA, which is an advantage when comparing the study populations. There was a high frequency of prematurity, low median birth weight, cystic fibrosis and no other syndrome was found, and also a few cases of twin birth; four characteristics were found typically in these study populations. 4 11 16 17

## Conclusion

In children with JIA, median time to full enteral feeding was 17 days and median LOH stay was 32 days. Low birth weight seemed associated with a longer hospital stay, and central line complications seem related to the duration with central line. Patients with a central line complication experienced longer hospital stay and longer time to full enteral feeding. The overall complication rate was high at 66% with the majority having a grade II- or III complication according to the Clavien-Dindo classification. We found no independent risk factor for the need of a reoperation. The small cohort may constitute a power problem in this study and further research regarding the included variables may reveal more potential predictors for the postoperative outcome.
